# Calendar

**DOI:** 10.1155/S1463924689000180

**Published:** 1989


					H. Stray Sample introduction valve for ICP-AES
the sample valve, and can be reduced by prolonging the
autosampler delay time. The concentration of the
measured elements would normally range from to 1000
and correction for the carry-over was only necessary in
special cases. The valve system has also been used with
other analytical programs where background correction
was used for the analysis of 20 elements with a total
exposure time of 20 s. Because of the valve system one
analysis could still be performed in 36 s. The flow rate of
Pump 2 was then reduced to 6 ml/min. Ifthe valve system
is to be used for programs with exposure times exceeding
20 s, larger loops must be installed.
References
1. WILLARD, H. H., MERRITT, L. L., DEAN,J. A. and SETTLE,
F. A., Instrumental Methods ofAnalysis (Wadsworth Publish-
ing Company, Belmont, CA, 1988).
2. HOLMBERG, M., Kemia-Kemi (Finland) No. 6 (1986),
554-556.
Calendar
MARCH
6-10 March 1989 Pittsburgh Conference 1989: Atlanta, GA,
USA. Contact Pittsburgh Conference, Suite 322, 12
Federal Drive, Pittsburgh, PA 15235, USA.
7-9 March 1989 Workshop: StatisticsforAnalytical Chemists:
Eastbourne, UK. Contact Christine Robinson, Statistics for
Industry (UK) Ltd, 4 Victoria Avenue, Knaresborough,
North Yorks HGF 9EU, UK.
21-22 March 1989 Research and Development Topics in
Analytical Chemistry: Dublin, Ireland. Contact Analytical
Division, Royal Society ofChemistry, Burlington House,
Piccadilly, London W1V 0BN.
30 March 1989 Analytical Atomic Spectroscopy in the Environ-
ment: Glasgow, UK. Contact Analytical Division, Royal
Society of Chemistry, Burlington House, Piccadilly,
London W1V 0BN.
APRIL
4-7 April 1989 RSC Annual Congress: Hull, UK. Contact
Gina B. Howlett, Conference Officer, RSC, Burlington
House, Piccadilly, London W1V 0BN. The Analytical
Division session will be on Process Analysis and Information
Management.
5-6 April 1989 Laboratory Science and Technology Show:
Cambridge, UK. Contact Curtis Steadman and Partners
Ltd., The Hub, Emson Close, Saffron Walden, Essex
CB 10 1HL, UK.
11-14 April 1989 VIIth International Symposium on Electro-
analysis and Sensors in Biomedical, Environmental and Industrial
Sciences: Loughborough, UK. Contact Dr A. G. Fogg,
Chemistry Department, University of Loughborough,
Leicestershire LE11 3TU, UK.
12-13 April 1989 Laboratory Manchester: Manchester, UK.
Contact Curtis Steadman & Partners Ltd., The Hub,
Emson Close, Saffron Walden, Essex CB10 1HL, UK.
19 April 1989 Chemometric Techniques in Clinical Chemistry
and the Pharmaceutical Industry: Salford, UK. Contact
Analytical Division, Royal Society ofChemistry, Burling-
ton House, Piccadilly, London W1V 0BN.
JUNE
25-30 June 1989 Eurosensors III and 5th International
Conference on Sensors and Actuators: Montreux, Switzerland.
Contact Eurosensors (Transducers '89), COMST S.A.,
PO Box 415, 1001 Lausanne 1, Switzerland.
JULY
30 July-5 August 1989 SAC 89: Cambridge, UK. Contact
Analytical Division, Royal Society ofChemistry, Burling-
ton House, Piccadilly, London W1V 0BN.
SEPTEMBER
11-13 September 1989 RSC/SCI/IOP Analysis Techniques
and Applications: Manchester, UK. Contact Mrs E. S.
Wellingham, Field End House, Bude Close, Nailsea,
Bristol BS19 2FQ, UK.
25-27 September 1989 Sensors and their Applications IV:
Canterbury, UK. The Meetings Officer, Institute of
Physics, 47 Belgrave Square, London SW1X 8QX.
25-28 September 1989 Third International Symposium on
Kinetics in Analytical Chemistry: Dubrovnik, Yugoslavia. Con-
tact Professor Gordana A. Milonovi(:. Department of
Chemistry, University of Belgrade, KAC PO Box 550,
11001 Belgrade, Yugoslavia.
26-28 September 1989 The British Laboratory Week:
Olympia, London. Including Laboratory 89, Computer
Aided Sciences, Bio 89, Medical Laboratory Sciences and
Analyticon. Contact Curtis Steadman and Partners, The
Hub, Emson Close, Saffron Walden, Essex CB10 1HL.
86

				

